# German Aerospace Center's advanced robotic technology for future lunar scientific missions

**DOI:** 10.1098/rsta.2019.0574

**Published:** 2021-01-11

**Authors:** Armin Wedler, Martin J. Schuster, Marcus G. Müller, Bernhard Vodermayer, Lukas Meyer, Riccardo Giubilato, Mallikarjuna Vayugundla, Michal Smisek, Andreas Dömel, Florian Steidle, Peter Lehner, Susanne Schröder, Emanuel Staudinger, Bernard Foing, Josef Reill

**Affiliations:** ^1^ DLR (German Aerospace Center), Institute of Robotics and Mechatronics, Muenchener Str. 20, 82234 Wessling, Germany; ^2^ DLR, Institute of Optical Sensor Systems, Rutherfordstraße 2, 12489 Berlin, Germany; ^3^ DLR, Institute of Communications and Navigation, Muenchener Str. 20, 82234 Wessling, Germany; ^4^ ESA/ESTEC, European Space Research and Technology Centre, Postbus 299, 2200 AG Noordwijk, The Netherlands

**Keywords:** robotics, planetary exploration, ARCHES demonstration mission, Martian Moons eXploration (MMX), Mobile Asteroid Scout (MASCOT)

## Abstract

The Earth's moon is currently an object of interest of many space agencies for unmanned robotic missions within this decade. Besides future prospects for building lunar gateways as support to human space flight, the Moon is an attractive location for scientific purposes. Not only will its study give insight on the foundations of the Solar System but also its location, uncontaminated by the Earth's ionosphere, represents a vantage point for the observation of the Sun and planetary bodies outside the Solar System. Lunar exploration has been traditionally conducted by means of single-agent robotic assets, which is a limiting factor for the return of scientific missions. The German Aerospace Center (DLR) is developing fundamental technologies towards increased autonomy of robotic explorers to fulfil more complex mission tasks through cooperation. This paper presents an overview of past, present and future activities of DLR towards highly autonomous systems for scientific missions targeting the Moon and other planetary bodies. The heritage from the Mobile Asteroid Scout (MASCOT), developed jointly by DLR and CNES and deployed on asteroid Ryugu on 3 October 2018 from JAXA's Hayabusa2 spacecraft, inspired the development of novel core technologies towards higher efficiency in planetary exploration. Together with the lessons learnt from the ROBEX project (2012–2017), where a mobile robot autonomously deployed seismic sensors at a Moon analogue site, this experience is shaping the future steps towards more complex space missions. They include the development of a mobile rover for JAXA's Martian Moons eXploration (MMX) in 2024 as well as demonstrations of novel multi-robot technologies at a Moon analogue site on the volcano Mt Etna in the ARCHES project. Within ARCHES, a demonstration mission is planned from the 14 June to 10 July 2021,^1^ during which heterogeneous teams of robots will autonomously conduct geological and mineralogical analysis experiments and deploy an array of low-frequency antennas to measure Jovian and solar bursts.

This article is part of a discussion meeting issue ‘Astronomy from the Moon: the next decades (part 1)'.

## 1. Introduction: robotics in future planetary missions

The exploration of the satellites of^1^ our solar planets to answer fundamental scientific questions has long been a desire of mankind. Scientists hope to better understand the origin of our Solar System and even to come closer to the answer of the question on how life was formed. Some moons of our Solar System are potential candidates to find life, such as Enceladus, Europa, or Titan [1]. Moons are also proposed to be used as gateways and entrance points for future human space missions. Concepts were proposed to build up even infrastructure on our Moon, like a Moon base [2] or a variety of different scientific instruments [3,4], describing for instance the use of a radio telescope on the far side of the Moon. However, reaching these moons is a very challenging task. In most cases, it would be too dangerous to deploy human missions, not mentioning the needed financial support for such an endeavour. Other problems arise when it comes to maintaining possible Moon bases in future missions. These bases are most likely not inhabited at all times; therefore, humans will not be able to maintain them the entire time and also not be able to conduct scientific experiments between theses times, particularly when instruments break and need repairs.

Robots have great potential to realize future planetary exploration missions. They help us reach solar objects and therefore enlarge the potential operational space, without putting human lives at risk. The use of this technology also helps to speed up mission cycles. However, with the use of robotics, new challenges emerge. While it is possible to remotely operate robotic systems if they are in Earth orbit or on the surface of Earth's Moon, it becomes more difficult if the robots are deployed further away. In such situations, long communication delays are introduced. Furthermore, due to radiation and low-communication bandwidth, directly controlling a robot becomes impossible.

The Mars missions with the rover ‘Curiosity', but also its successor ‘Perseverance', which was launched together with the novel Mars helicopter ‘Ingenuity' in 2020, and last but not least the ESA Exomars Rover planned to be launched in 2022, will raise the request for a higher degree of autonomy in operations than earlier missions. The physical distance to Mars leads to communication round-trip times between 8 and 40 min, depending on the current constellation. Thus, the requirement for local intelligence on board the robots will increase in order to enable more and more complex autonomous operations, both to ensure the safety of the systems and to increase the scientific output by speeding up the mission. The Mars helicopter, for example, needs to operate fully autonomously during flight as its envisioned flight time is below the minimal communication round-trip time to Earth.

Within several activities of different national agencies, e.g. NASA [5], JAXA [6], ROSCOSMOS [7], CNSA [8] and ESA [2,9], the Earth Moon is in focus for many upcoming missions. During the activities of ROSCOSMOS, after the LUNA25–27 missions planned for the next years, the LUNA28 mission aims to land on the lunar South Pole and explore either the Schrödinger Basin and/or land in the vicinity of the South Pole Aitken Basin. Moreover, the Indian Space Research Organisation (ISRO) has set up the Chandrayaan programme, a three-step exploration roadmap for the Moon with the ambitious goal to launch in 2024 an on site sampling mission to the pole. The lunar South Pole is also the target of the ESA Heracles project [10] and also of EL3 (European Large Logistic Lander) mission. Both projects overlap significantly in terms of target regions and mission profiles [3] and the planned repeatability as well as the high payload capability of the EL3 lander allows the idea of multi-robot missions. Meanwhile, during different EL3 scenarios, the mission set-up is focused on three aspects:

— scientific output and sample return capabilities;
— investigations for ISRU (In-Situ Resource Utilisations) and technology demonstration also for scientific instrumentations; and
— demonstration of capabilities to provide cargo delivery to the lunar surface in the frame of the NASA Artemis programme.

Besides the lunar surface activates, orbiting stations are also of special interest. The CIS Lunar Station, renamed ‘Lunar Gateway', is planned to orbit the Earth's moon. It will be occupied by humans for only one month of the year. This is why robotic operations inside and outside the station will have to increase its functionality and capabilities. This station shall serve as a signal relay to surface activities, but could also host operators and supply materials, which then leads to different communication capabilities and thus robotic operational concepts.

Although these semi-autonomous functionalities help to tackle many problems of robotic planetary exploration, they are not able to replace a technical skilled human in the loop. This makes it difficult to have missions, which are beyond a robust enough communication link to execute basic commands and monitor the robots status, as would be the case in missions to our ice moons of Saturn and Jupiter [1]. As a result, it is necessary to develop technologies for a fully autonomous robotic system that do not need a technical human in the loop to perform its scientific tasks. A team of scientists should get reports from time to time from the robot about the collected scientific data and findings and execute high-level commands on what the robotic system should do next.

The aim of this paper is to give an overview of the past, present and future robotic missions for lunar planetary exploration, in which DLR and several of its direct partners are involved. The paper demonstrates how a variety of technologies were developed in a variety of missions and reused in ongoing ones with the goal of developing an autonomous robotic system for future lunar missions. These technologies also include a set of components to take scientific measurements and perform *in situ* analyses.

The paper first gives an overview of two past DLR missions, which are relevant for robotic lunar exploration. This includes the Mobile Asteroid Scout (MASCOT) mission and also the Robotic Exploration of Extreme Environments (ROBEX) missions. The section ends with a summary of lessons learned from these missions, which are important for upcoming projects. The next section deals with present and future missions. Here, we describe our ongoing ARCHES project and also the future mission Martian Moons eXploration (MMX), which will send a rover to the Martian moon Phobos.

## 2. Past steps to autonomous robotic space missions

In order to come closer towards the goal of developing robotic technologies for future lunar missions, DLR participated in a variety of different missions with its partners. This section describes two of them, the MASCOT mission and the ROBEX mission. Both helped to develop key technologies which are important for future fully autonomous lunar scientific missions.

### (a) Mobile Asteroid Scout

**Figure 1.**
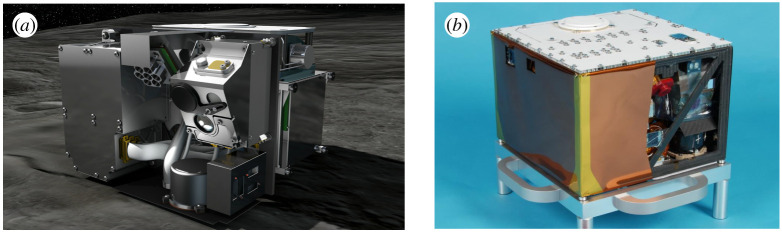
(*a,b*) Artist impression of the MASCOT lander on Ryugu, photo of the MASCOT lander flight model; dimensions: 29 × 28 × 21 cm, weight: 10 kg. (Online version in colour.)

The Hayabusa2 mission demonstrated impressively the capability of current space technologies. JAXA conducted complex and dangerous touch-and-go manoeuvres with its spacecraft to take soil samples from the asteroid Ryugu. Unlike the first failed attempt in 2005 with Hayabusa1 to the asteroid Itokawa, the one in 2019 was a complete success [11,12]. Hayabusa2 also hosted a small 28 × 29 × 21 cm landing module, which was supposed to land on the asteroid and conduct several scientific measurements. DLR contributed by developing a locomotion and uprighting mechanism for its MASCOT landing unit. This mechanism rotates the module in the correct upright position in order to perform its scientific task. The module landed on the surface of the asteroid on 3 October 2018. The lander conducted a first uprighting attempt using its MASCOT autonomy manager without interaction from Earth. As the position and orientation of the spacecraft was fixed to keep communication links to Earth and point its solar arrays to the sun, communication with the lander was lost right after touchdown due to the asteroid's self-rotation (period of 7.6 h). The lander needed to take action on its own to save time for carrying out scientific tasks. When communication returned, the lander reported to have uprighted and completed a first run of scientific experiments successfully. Analysis of the sensor data on the ground showed that the lander was actually oriented upside down and needed additional commands from the ground. The on-board GNC sensors probably failed due to very dark albedo of the asteroid and higher distance to the soil than expected. The asteroid was very rocky and not sandy as expected during mission planning years before. The autonomy manager was interrupted by a command from ground control and a relocation attempt was initiated. MASCOT successfully relocated on the asteroid's surface and came to rest at nominal orientation, thereby enabling operation of the scientific instruments *in situ* [13]. These exciting hours of operation showed the superiority of combined autonomous and human-in-the-loop operation, as the overall mission could be completed successfully. Operating the lander only by telecommands was not an option due to time delays of 16 min for one communication way and the limited lifetime of the lander due to its battery capacity. This small robot unit, which was built mainly with COTS parts, provides a high payload capacity for scientific equipment (3 kg) of a total mass of MASCOT (10 kg) [14]. The scientific payload consisted of a science camera, a magnetometer, an infrared radiometer and a near-infrared hyperspectral microscope. The instrument set-up was chosen considering the mass and energy budget and to support the soil sample part of Hayabusa2 mission by *in situ* measurements and investigation on the asteroid's components. It has proven its functionality in deep space and increased the used DLR technologies and scientific instrumentations to TRL 9 (figure 1). The idea of using a payload box to carry a different kind of scientific instrument proved to be very promising and was reused for future mission scenarios.

### (b) Robotic Exploration of Extreme Environments mission

**Figure 2.**
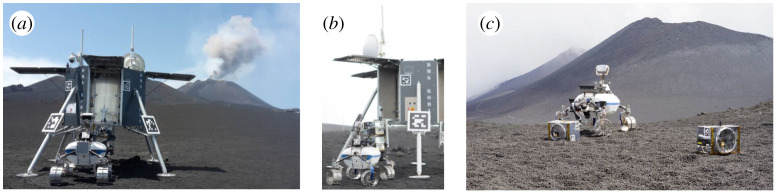
(*a*–*c*) ROBEX experiments at a Moon analogue site on Mt Etna: autonomous deployment of an array of seismic sensors housed in modular payload boxes with our Lightweight Rover Unit (LRU) rover prototype [15,16]. (Online version in colour.)

The Robotic Exploration of Extreme Environments mission was a Helmholtz Alliance research project from 2012 until 2017 with the aim to enhance Germany's strength in robotics for extreme environments such as deep sea and space exploration [15]. During the project, the goal of increasing autonomous robotic capabilities for both application domains was the common technological research goal, meanwhile the focus was also to bring research into operation, increase technology readiness and show the functionalities during test demonstrations in realistic environments. Therefore, the deep sea domain has executed a demonstration (October 2017) with the research vessel ‘Polarstern' in the Artic region (Svalbad) and the space domain has performed the ROBEX analogue mission during June/July 2017 on Mt Etna, which has brought together several DLR institutes with other German research organizations to demonstrate autonomous robotics capabilities in the hazardous deep sea and outer space environments. Within this project, novel technologies have been demonstrated in the context of planetary exploration through the deployment of a seismic network array performed in complete autonomy by our Lightweight Rover Unit (LRU, [16]) in a Moon analogue environment on Mt Etna, an active volcano in Sicily, Italy in 2017. Figure 2 shows our LRU at the Moon analogue site during the ROBEX field tests. The space mission scenario in ROBEX was inspired by the idea that seismic experiments are fundamental for almost every planetary mission to increase the understanding of the inner body structure, e.g. the inner crust model and thickness of crust layers, but also to analyse the seismic activity of the body and to detect meteorite impacts on the body. The two performed mission sequences in ROBEX were based on the active and passive lunar seismic experiments (inside the Apollo Lunar Surface Experiments Package) performed during Apollo lunar missions [17], meanwhile ROBEX was capable of showing the deployments performed with one partly autonomous operated robot. The seismic instruments, in Apollo only geophones, in ROBEX full three-axis seismometers, have been implemented inside the flexible payload carriers—‘Remote Units', based on the design of the MASCOT instrument box. The RU30 (Seismic Remote Unit 3 kg) was a reduced functional engineering model version of the RU10 (Seismic Remote unit 10 kg), which was focused on unifiable components, while the RU30 was required by ROBEX to deploy the passive seismic array triangle in approximately the same dimensions as the previous version of Apollo 17. The Etna site was chosen as ideal, since regular seismic events due to tectonic movements in this region are happening in a focal deep depth of approximately 600 km, which is similar to the depth of deep lunar quakes observed during the Apollo mission. Furthermore, the seismic activity is unique due to the fact of being on an active volcano with continuous tremor activity and its location on tectonic borders. Moreover, the site provides fresh volcanic regions with geological highly interesting features and diverse regions with different soil and surface shapes, which make Mt Etna ideal for exploration testing missions.

### (c) Lessons learnt

The previous sections described briefly the idea of modular scientific payload carriers and robotic modules used in different missions and analogue field tests. Especially in the preparation and retrospective of the past ROBEX, the analogue campaign has produced a large number of findings and lessons. The most important ones, which complement the results of the campaign and which can be transferred to other campaigns in a generalized form, are collected below.

The overall duration estimates for tests and tasks, based on the experience with previous laboratory and even field tests, were significantly underestimated.

— Factors contributing to these findings are the unstructured terrain and environmental factors of the remote site where especially the access time of the personnel on site has dominant time influences. These factors are also considered as the main reason for conducting an analogous campaign to identify exploration-like factors in the mission concept.
— Other factors were not of lunar analogue origin and range from unfavourable weather (sunny and calm scenes obscure the fact that Etna is a high alpine region with rapidly changing visibility and wind conditions); furthermore, access of tourists disturbed the test runs (the test site is a World Heritage Site and exclusive access to the test teams was not granted by the authorities).

Most of the total delays were a mixture of both groups, and some were indirectly caused by them (e.g. empty batteries after unplanned dwell times), making it difficult or almost impossible to break them down into relevant and irrelevant delays. Although cumbersome, a better event and ‘side factor’ tracking could be worthwhile. When planning the field test campaign, a large schedule for contingencies should be provided.

A clear definition of a catalogue of criteria for mission success was useful and is recommended. However, campaign organizations need to be aware and acknowledge that different ideas of `mission success' could or should remain and need to be balanced. For example, a certain amount of technology demonstrations aims for mission tasks with high degree of automation or autonomy, which might be acceptable for mission engineers or project scientists. Meanwhile from a robotics point of view, the technical verification and the demonstration of repetitions of, e.g., a seismic experiment might be sufficient from an engineering point of view to show the reproducibility of a process, but might not be sufficient for a scientific evaluation of the geophysical measurements. It is recommended to record and archive data (structured and unstructured) as ‘by-products’ beyond the immediate needs of the campaign. For example, the recordings of the field weather station were later used to determine wind conditions in conjunction with seismic recordings as a contribution to the InSight mission. Furthermore, raw data recordings of the robotic procedures have made us capable of recalculating full processes using simulation later in the laboratories. Especially during SLAM and navigation experiments, these data recordings and simulation capabilities have allowed significant improvement of the algorithm with real-world data, which also has been made available to the community [18]. Important technical findings were the weakness of WIFI signal of the data link between lander, payload carrier and rover when they were close together. The lander structure with unfolded solar cell fields obstructed the line of sight to the antennas mounted above and shielded the rover and the instrument carriers. Communication signal strength and link availability for very mission-critical operations in close proximity must be evaluated very carefully, especially considering WIFI protocols, which are made for indoor use and improve with reflections.

The lessons learned from MASCOT as a real deep space flight mission is manifold and exceeds the terrestrial experiences by far. One obvious additional aspect is the mission duration. The personnel of the development team changed quite a lot beginning from 2010 to the touchdown in October 2018. The operations team still needs to know all system details and engineering solutions to be prepared for all kinds of unexpected circumstances. In particular the option to reconfigure the system behaviour is crucial. In the beginning, the concept for the descent was to touch down and come to rest due to sandy and energy-dissipative soil. However, the first camera images in summer 2018 revealed that the asteroid's surface did not consist of sand at all, but is covered by a large number of big boulders, which are spread uniformly over the surface. As a result, it was not possible to choose a landing site based on the previously defined criteria. This is why a team hierarchy is needed to be able to take quick and carefully considered decisions. In the end, it was necessary to decide on how to proceed during the mission based on a small number of data. Some instruments were able take measurements even with the MASCOT orientation upside down, while others were not able to collect data at all. With human operators taking many unforeseen parameters into account, the command for relocation saved the overall mission goal. Nevertheless the autonomy manager was still needed to perform the sequences of scientific operations by sending only a few high-level commands. During the preparation time, it is hard to imagine that the only data that will be available during the mission are a few status flags. All other data products need even more time to be transferred and analysed, through the very limited communication channel. Half an hour was lost before seeing the effect of a single command, which is a long time considering the overall lifetime of roughly 17 h due to battery lifetime. In addition to the planned scientific operations, the design of the autonomy manager needed considerations regarding battery lifetime and thermal constraints since the round-trip time was several hours. The power drain from the battery was optimized to extend the lifetime and also to control the temperature inside the electronics box. The transfer of data to the spacecraft needed strict planning as the connection was dependent on asteroid rotation. Another aspect was the development time of only four years to set-up a lander for such a harsh environment. Unit tests sped up developing processes, but still an overall system test was needed in vacuum and thermal environment to make sure that EMC, thermal and power conditions are within the expected range. Due to the fact that the installed instruments are very sensitive, calibration and reference measurements need to take place. Partly this was conducted during cruise phase because the launch window was fixed.

## 3. Present and future works towards planetary exploration: ARCHES and Martian Moon eXploration

In this section, we describe our current ongoing and future efforts in order to build up scientific robotic systems for lunar missions. The first part illustrates the ARCHES mission, which has the task to develop and demonstrate key technologies for a heterogeneous robotic team. The second part deals with the MMX mission, which has the aim of sending a semi-autonomous robot to the Martian Moon Phobos.

### (a) ARCHES

The contribution of the ARCHES project [19,20] to DLR's roadmap towards future lunar missions is the development and demonstration of *cooperative* behaviours among a *heterogeneous* team of robots. The team, operating in semi- and full autonomy, becomes thus a complex system able to explore, perform scientific analyses and deploy sensor networks in a way that is difficult or impossible to accomplish otherwise. Cooperation of robotic agents allows, in contrast with independent single-agent operations, to fulfil common mission goals while maximizing efficiency, through task parallelization, and robustness, through functional redundancy. Heterogeneity spreads the complexity of large-scale operations to a variety of specialized agents, which can operate more efficiently and robustly. Furthermore, heterogeneous agents benefit from their complementary nature: flying vehicles, not limited by the ground traversability, augment the perceptive reach of their ground counterpart which, in turn, can physically and safely interact with the environment. Prospects of employing flying vehicles for future planetary exploration are indeed very concrete: as demonstrated by NASA's Ingenuity Mars helicopter, to be deployed by the Perseverance rover in 2021, many efforts are being made to prototype and test Unmanned Aerial Vehicles (UAVs) for planetary environments with thin to no atmosphere [21].

While in ROBEX only a single robot was operating autonomously to deploy seismic boxes, in the context of ARCHES the robotic team comprises two Lightweight Rover Units, LRU1 and LRU2, and the ARDEA UAV acting as a fast scout. LRU1 is specialized in the analysis of rocks and three-dimensional reconstruction thanks to its multi-spectral and high-resolution stereo cameras. LRU2 instead is specialized in manipulation and therefore will be employed for sample collection tasks and deployment of scientific instrumentation. An additional contribution of the ARCHES project is the expansion of the concept of modularity beyond the scope of robotic agents by also including the sensors, which are otherwise traditionally related to specific robots. Standardized payload boxes carry communication and power infrastructures as well as scientific instrumentation to conduct *in situ* experiments. This concept is inherited from the DLR's experience with MASCOT and facilitates the collaboration within robotic teams towards scientific operations.

The major scientific goal within the ARCHES project is the feasibility demonstration of deploying, through a network of autonomous or semi-autonomous robotic agents, a low-frequency antenna array (LOFAR) on a Moon-like surface. Aside from this major task, the robotic network will cooperate towards the autonomous exploration of an unknown environment with the purpose of detecting and analysing rock samples to determine their chemical composition. A large-scale demonstration mission is planned for the evaluation of the aforementioned tasks. The mission goals are scientifically and technologically aligned to the current goals of DLR's next exploration activities. The robotic sample-return and also *in situ* analysis capabilities are requested in lunar mission scenarios, as described above, but also implemented in the MMX mission design. The use of scientific imagers, such as cameras for visual, spectral and thermal imaging, combined with *in situ* instrumentation to perform Raman spectroscopy, to be performed on-board the MMX rover [22,23], or Laser-Induced Breakdown Spectroscopy (LIBS), greatly increase the scientific outcome of these missions.

Considering that the location and environmental context of the locations selected for sampling and data acquisition is highly relevant for scientific interpretation, it is very important for the sample fetching system to localize itself and map the sampling area precisely for subsequent investigation. The SLAM (simultaneous localization and mapping) capabilities of the robotic systems play an important role for that goal [24,25]. The robot needs to be able to localize itself in real-time in GNSS-denied environments using its on-board sensors and computation resources. Since flying robots such as drones are also used in the ARCHES mission, the fusion capabilities of lower resolution drone data from a bird's-eye perspective with ground-based, high-resolution maps increases the ability to combine different maps and data acquisitions to build up a common model of the mission area. In this article, we focus on the scientific and technological aspects regarding the acquisition of scientific measurements, whereas we refer to [20] for an overview of the individual technological aspects required for autonomous robotic operations. The following sections summarize the two principal tasks to be performed as part of the ARCHES demonstration mission: a sample-return mission performed by highly autonomous robots and the installation and maintenance of infrastructure elements: deployment of a distributed antenna array for low-frequency radio observations (LOFAR).

#### (i) Sample return mission

The focus of the first two mission scenarios is on the autonomous and semi-autonomous cooperation within a heterogeneous team of robots equipped with *in situ* instrumentation such as LIBS spectrometers and multi-spectral cameras. LIBS is a technique for fast *in situ* geochemical and mineralogical analysis and is particularly sensitive to all kinds of metals and hydrogen, where the latter could serve as an indicator for volatile water in the lunar regolith. In addition, other robotic assets like manipulation and sample collection skills and the complementary abilities of the flying drone [26,27] will demonstrate the benefits of a heterogeneous team of robots to succeed in the overarching mission scenarios (see figure 5).

**Figure 3.**
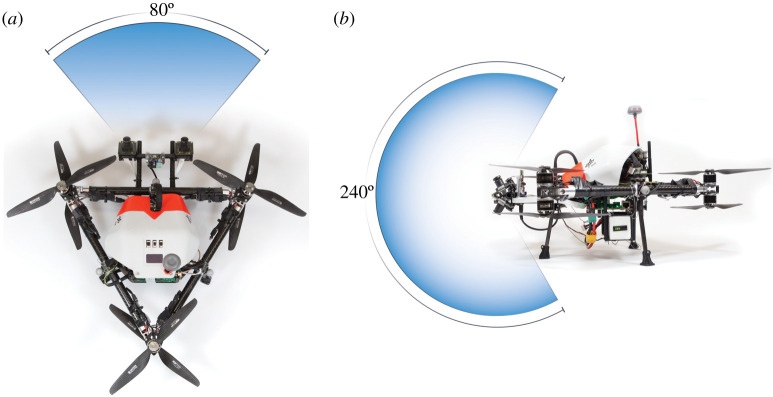
(*a*,*b*) Scouting drone ARDEA [27] with the field of view of its stereo-vision multi-fisheye camera system. (Online version in colour.)

**Figure 4.**
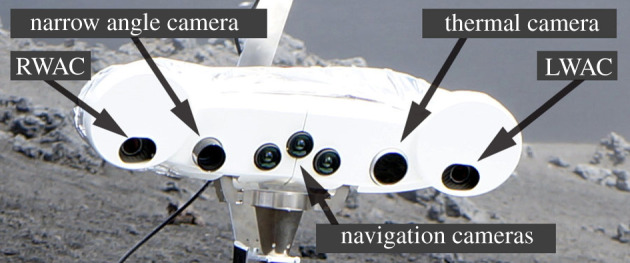
ScienceCam [20] with its left- and right wide-angle cameras (LWAC and RWAC) featuring colour- and narrow-band spectral filter wheels. (Online version in colour.)

The drone will explore the area first and scout for points of interest that have been defined by scientists based on satellite images. A flying robot is able to cover a larger area of interest faster and can traverse terrain which might be challenging for the ground rover (figure 3). During the exploration by the drone, the scenery will be mapped and visual information of particular regions of interest will automatically be extracted and sent back to the mission operation centre, where scientists and operators define the actions for the next set of robots. Although our drone prototype has been developed for environments with a dense atmosphere, the autonomous navigation functionalities are designed to be used in any planetary environment, including the surface of the Moon. Furthermore, our drone is made up of two parts: the navigation stack and the propulsion frame [27]. The propulsion part can be exchanged based on the environment where the drone is to be deployed. On surfaces with thinner atmosphere, this might be larger propellers, whereas in environments which do not have any atmosphere thrusters might be used [21,28]. Independent of the choice of propulsion frame, most components of the navigation frame stay unchanged, like the estimation of the current position of the drone. A high-level autonomous planer will suggest the next actions and execute them if there are no interactions from scientists and operators. Once an area of interest has been spotted the LRU1 is sent out for further inspection. The rover features a mast with an array of different kinds of scientific sensors (figure 4), such as a thermal camera, high-res camera and spectral filter wheeled with 15 different kinds of filters.

All of these sensors are helpful in order to identify different geological targets which are identified by a team of scientists and marked for later collection by LRU2. We refer to [20,29] for further details on the geological sampling scenario. A close cooperation with ESA research for the shared autonomy and tele-operational scenario is in place with the Multi-Purpose End-To-End Robotic Operation Network (METERON programme) at different ESA locations [30]. It will test the feasibility of remotely manoeuvring a mobile robot and controlling a manipulator for sample collection, as already performed by astronaut Luca Parmitano in a series of experiments denominated ANALOG-1, where a mobile robot was controlled from the International Space Station (table 1).

**Table 1.** Science Traceability Matrix for the ARCHES Demonstration Mission.

scientific objectives	technological mean	systems/instruments involved	technical requirements and instrument specifications
observation of Jupiter and solar radio bursts	— deployment of a low-frequency array radio telescope (LOFAR) — calibration of the low frequency radio array — localization of LOFAR antennas	— self-unfolding short dipole antennas embedded in payload boxes — radio-CPT (communication, positioning and time-synchronization) — ARDEA: inspection potential deployment areas — LRU2: manipulation of sensor boxes	— precise localization of each antenna of the array by radio communication, positioning and timing system (Radio-CPT). Positioning accuracy in order of 1/10 wavelength — carrier frequency between 20 MHz and 30 MHz — Deployment on an area of 100 × 100 m — number of array elements 3
elemental analysis of rock and sand samples	— LIBS (Laser-induced breakdown spectroscopy) — multi-spectral imaging (ScienceCam)	— LIBS spectrometer embedded in payload boxes — ARDEA: inspect potential areas regardless of traversability — LRU: inspect terrain with spectral cameras (ScienceCam) to spot interesting targets — LRU2: manipulation of payload boxes	— LIBS Weight approximately 1 kg — Nd: YAG laser wavelength 1064 nm with 8 mJ and 6 ns pulse — LIBS spectrometer covers a range of wavelength of 550–770 nm — detect hydrogen, silicon, calcium, sodium and potassium and more — ScienceCam: 9 narrow-band filters (wavelengths of 450, 440, 660 nm and FERRIC filter set) mounted on pan-tilt unit

#### (ii) Deployment of low-frequency antenna array: autonomous setup of scientific instruments

A forward-looking vision of astronomers worldwide is the operation of a large-scale radio-telescope on the far side of the Moon to observe the sky in the radio-frequency (RF) range of 1 MHz to 40 MHz with unprecedented quality. Observations in this frequency range, and free of interference from Earth's ionosphere, enables insights into the dark ages of the Universe, the magnetospheres and space environments of possibly habitable exoplanets and our sun [4,31,32].

**Figure 5.**
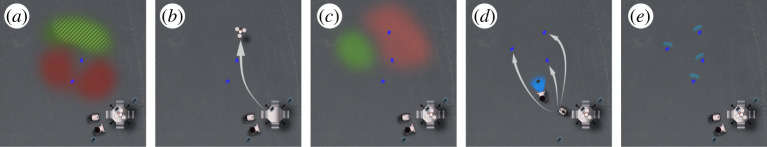
Graphical summary of the sequence of actions for LOFAR deployment on a Moon-like environment as part of the ARCHES demonstration mission. From top left to bottom right: (*a*) mission control denotes potential locations for the antenna array; (*b*) a drone scouts the target locations to assess the feasibility of deployment; (*c*) mission control defines final locations for the antenna array; (*d*) ground robots are sent to position the LOFAR boxes; (*e*) the antenna array is deployed and calibrated. The different grey marked areas (red and green online) denote optimal and sub-optimal locations for the incremental deployment of the next array elements, white arrows denote the movements of a robotic unit and dark (blue) markers are LOFAR boxes. The background image is a top-down view of the test site on Mt Etna, Sicily, where the ARCHES demo mission will take place. Note that the image is not drawn to scale but serves only for illustration purposes. (Online version in colour.)

Multiple concepts for a lunar radio-telescope exist, and are mostly tethered antennas robotically deployed around a lander. In contrast with these concepts, the goal of the ARCHES LOFAR experiment is to demonstrate the feasibility of autonomously deploying an array of untethered antennas with a team of robots, thanks to a precise localization of all array elements based on a novel radio communication system. Figure 5 visualizes different parts of the mission from top left to bottom right. Scientists and mission operators define the array aperture and its placement on the lunar surface, and the first antenna element is autonomously deployed by a robot. Green shaded areas in figure 5 indicate optimal placement areas for the next array element based on radio communication coverage prediction, the topography and environmental condition, and the predicted resulting LOFAR precision. Red shaded areas indicate suboptimal regions for placement. The operator can still place an array element there, yet the operator becomes aware of a resulting sub-optimal solution. In many cases, the terrain is possibly unexplored in detail, and only images from orbiter with meter-level accuracy are available. Hence, the placement area must be re-mapped on-demand to determine traversability. In ARCHES, we use a drone as part of a heterogeneous team of robots to accomplish this task; see the upper middle image in figure 5. In a real lunar scenario, a hopper or a dedicated orbiter with very high spatial image resolution but very small field of view could be used. Once all deployment areas are explored, one rover successively fetches all antenna elements from the lander and places them on the ground; see the lower left image in figure 5.

**Figure 6.**
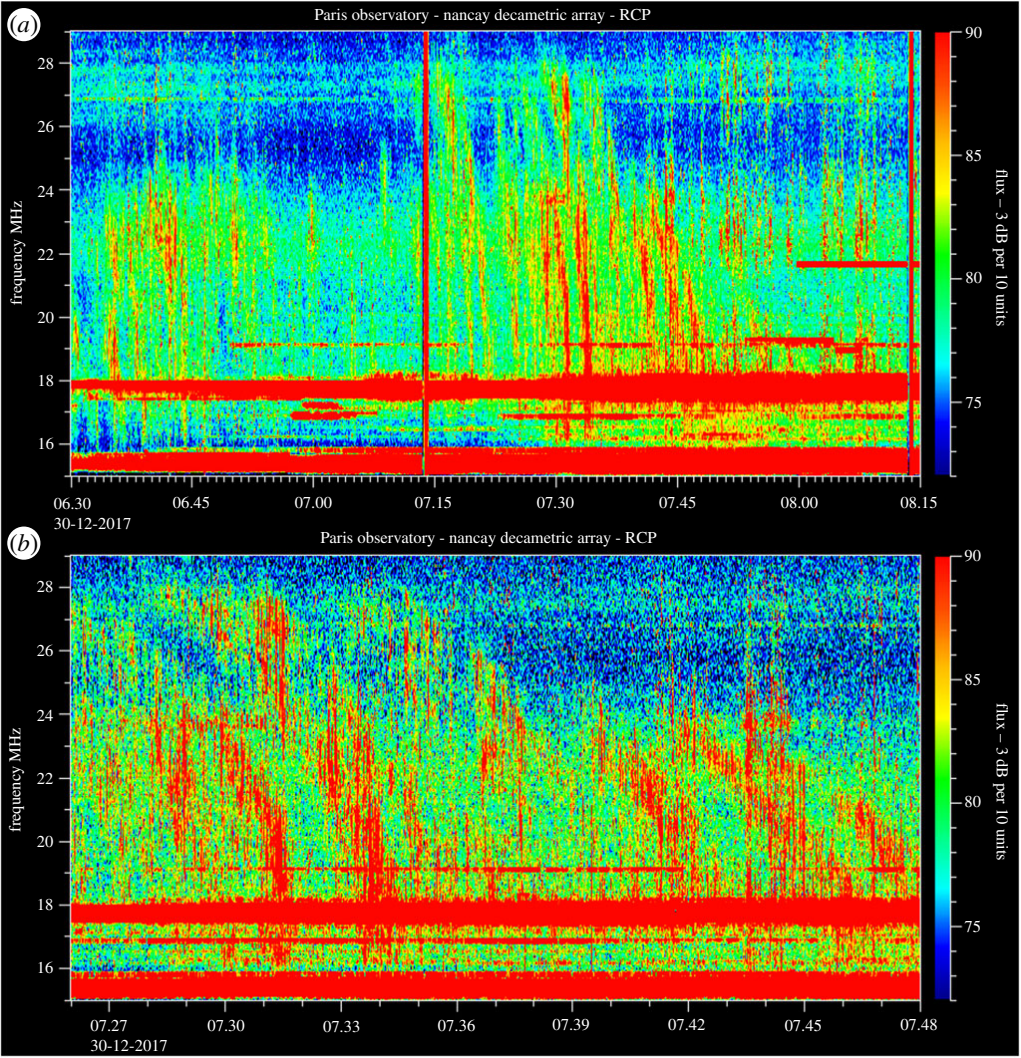
Illustration of time scales of radio solar bursts variations in 16–28 MHz VLF range during 2 h (*a*), with a zoom of 20 mn on one event (*b*) (courtesy Paris Observatory Nancay Decametric Array). During the ARCHES campaign, after VLF functional proof of concept validation, we plan to measure such variations and track them in space and time. (Online version in colour.)

**Figure 7.**
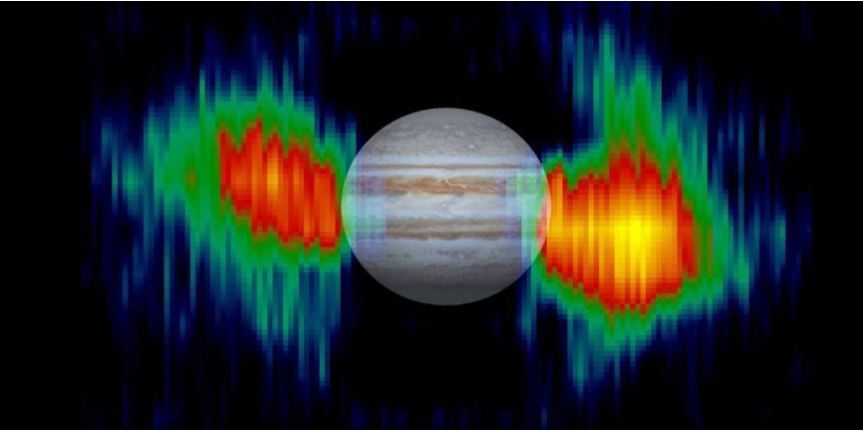
Image of Jupiter's radiation belts mapped from 13.8 MHz radio emission measured by the U.S. Cassini orbiter in January 2001 during its flyby of the planet. A superposed telescopic image of Jupiter to scale shows the size and orientation of the belts relative to the planet. Interpreted as synchrotron radiation, the emission delineates a doughnut-shaped region surrounding Jupiter where electrons moving near the speed of light radiate as they gyrate in the Jovian magnetic field. (Image courtesy NASA/JP). A lunar VLF radio interferometer can resolve the lobes and track the spatio-temporal variations of the lobes, in particular during Io induced radio bursts. (Online version in colour.)

In parallel, a second rover uses its visual navigation system to localize three array elements and the lander jointly, to determine the location and orientation of the array with respect to the lander and, hence, a global coordinate frame. Finally, all robots leave the area of the antenna array, and we use our novel radio communication, positioning and timing system (Radio-CPT) to precisely estimate the array geometry down to the decimetre-level based on time-of-flight measurements of radio communication packets [33,34]. Once localized, a self-unfolding short dipole is released and RF signals at 20 MHz carrier frequency are received, sampled and communicated to the lander for further processing. Processing those RF samples includes filtering, waterfall diagram calculation with different integration times and beam-forming/steering. Phase calibration of the array is important, and in ARCHES we use a dedicated calibration transmitter with a known location in the far-field of the array. In a real scenario, fixed radio sources in the sky can be used to calibrate the array. In addition, we use a dedicated low-frequency transmitter placed in a different location to emulate a low-frequency radio source such as the sun or radio bursts from Jupiter for proof-of-concept array operation. After this, we shall assess the prospect and sensitivity for acquiring solar (figure 6) radio bursts from these antennas deployed on the ground in a configuration similar to the lunar case, considering the radio electric properties of volcanic ash soil or lunar regolith. Additional measurements will be conducted on Jupiter (figure 7) that can outshine the quiet Sun during its Io-B bursts [35,36]. Jupiter will be well placed for observations during the Etna ARCHES campaign in June 2021.

Our antenna elements are based on the payload box design of MASCOT, developments and lessons learned from ROBEX in terms of robotic manipulation and new developments within ARCHES. Four boxes contain an off-the-shelf wireless communication system for back-up and data transfer, a GNSS receiver to determine ground-truth position to validate our Radio-CPT system, the Radio-CPT system comprising an off-the-shelf mini-computer and software-defined radio (SDR), and another off-the-shelf SDR with low-noise amplifier and antenna for low-frequency RF signal reception. Three additional boxes without the low-frequency receiving SDR are used to transform the array into a global coordinate frame. Precise localization is key for the array. Traditional visual navigation for robots is prone to drift in their estimation, resulting in increasing estimation biases for longer traverses. Hence, we use our Radio-CPT system to determine the array geometry accurately and jointly use results from visual navigation and radio-localization to obtain global information of the array geometry. In principle, the positioning accuracy of individual antenna elements should be in the order of a tenth of the low-frequency RF signal's wavelength, or even better. Residual positioning errors can jointly be taken into account during the phase calibration process.

### (b) Martian Moons eXploration

Owing to the success of the MASCOT joint development and cooperation between DLR and CNES, a follow-up operation with both parties contributing equally has been planned for the MMX mission [15] by JAXA. Again, JAXA will conduct the sample-return part of the mission and the DLR/CNES mobility system with a mass of up to approximately 29 kg will explore the Martian moon Phobos. The mission is scheduled to be launched in 2024 with the touchdown for the rover on Phobos being planned in 2026.

**Figure 8.**
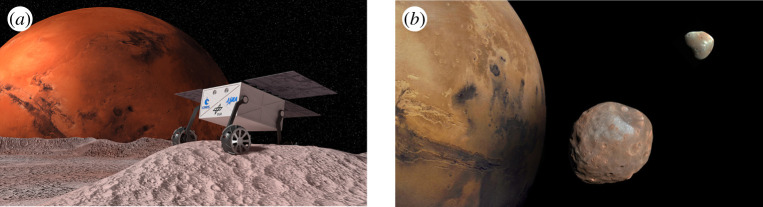
(*a,b*) MMX Rover (Credit CNES); Mars, Phobos, Deimos (Credit: NASA); at PDR status, the Rover body is cuboid with dimensions of 38 cm × 23 cm and a weight of 29 kg. (Online version in colour.)

The scientific goal of the MMX mission aims to distinguish the origin of the Martian moons Phobos and Deimos and increase knowledge on the Mars constellations and the knowledge of the evolution of the universe (figure 8). Sample-return and specific analysis of the surface material is a key success criterion for such scientific missions.

The objectives of the MMX rover are to perform terrain assessment to reduce the risk for the spacecraft landing approach and explore the unknown surface of Phobos. In addition, the rover also acts as a platform for scientific and technology demonstrations. In this regard, DLR is working on the locomotion and control subsystems of the rover. Furthermore, DLR will perform an autonomous navigation experiment on Phobos with the MMX rover as a technology demonstration. Autonomy is needed as teleoperation of the rover is hard because of the long communication times between Earth and Phobos as well as the short rover mission duration. For these activities, the experience and lessons learned from past missions and activities such as the MASCOT mission and the ROBEX mission play a vital role.

Similar to the instrument set-up of MASCOT, one of the primary goals of the MMX rover is to characterize the unknown components of the celestial body. The rover carries a RAX Raman spectrometer, miniRAD radiometer, navigation cameras and wheel cameras to analyse the wheel print on Phobos soil. The rover will be deployed at an altitude less than 100 m and will have to upright itself autonomously just as MASCOT needed to. Communication will not be established before unfolding its solar arrays and this definitely needs to be conducted when the rover is in nominal orientation. Owing to mass limitations, it is likely that there will be a preloaded passive mechanism to unfold the array such that it is not possible to pack the array back and rotate the legs again for a second uprighting attempt. Commanding the rover will be very challenging due to communication delays and many interruptions. It is not sure whether the spacecraft will stay at Phobos or go into a Mars orbit. This is why a lot of autonomy will be needed for navigation as well as overall mission planning. The rover needs to have very good energy management as the solar array is sufficient to heat and recharge the batteries but not sufficient to stay active during a full Phobos day.

The solution being developed for autonomy is based upon the years of research and development experience in developing autonomous navigation algorithms for space exploration scenarios. The effectiveness of such an autonomous navigation solution for exploration has been demonstrated through previous activities especially at the ROBEX space demo mission using the LRU. However, these navigation algorithms were not optimized to run on limited computational hardware, as is required for the MMX rover. In addition, the uncertainty and lack of detailed information on Phobos terrain, the low gravity on Phobos as well as the rover's limited power and sensor constraints make it a more difficult problem. If successful, the MMX rover will be the first rover to operate on and explore Phobos.

## 4. Conclusion and outlook

In this paper, we have presented an overview of past, present and future missions where DLR developed fundamental core technologies towards increased robotic autonomy in scientific planetary missions. We discussed our contribution to two actual space missions as well as two large-scale test campaigns in a Moon analogue environment, presenting both our past experiences as well as ongoing preparations for future missions for each of the two categories. This allowed us to highlight how the lessons learned from the past shape our current efforts. Specifically, we described the involvement of DLR in two missions led by JAXA and targeted to the exploration of small bodies: Hayabusa2 with the MASCOT and MMX with the MMX rover. We showed how the core technologies and the lesson learnt from MASCOT inspired a variety of other DLR activities such as the ROBEX demonstration mission and the development of the MMX rover itself. Within MMX, the concept of a deployable mobile sensor box evolved into a small rover thanks to the addition of a wheel locomotion subsystem. Within ROBEX, and by extension in ARCHES, this concept evolved into standardized and self-contained payload boxes that can be manipulated by any rover and carry arbitrary sensors, whereas in ROBEX, a single Lightweight Robotic Unit deployed an array of seismic instruments; in ARCHES, now planned for the summer of 2021, a team of heterogeneous robots will autonomously deploy sensor boxes for the mineralogical analysis of rocks as well as to form an array of low-frequency antennas for the observation of solar and Jovian bursts, in preparation of future autonomous and unmanned scientific lunar missions.

## Footnotes

1 The ARCHES demonstration mission was originally scheduled for 2020 but will now be shifted to 2021 due to the COVID-19 pandemic.

## Data accessibility

This article does not contain any additional data.
